# Skills and practices of pharmacy staff for dispensing of drugs with fiscalized substances in drugstores and pharmacies

**DOI:** 10.11606/s1518-8787.2021055003103

**Published:** 2021-06-25

**Authors:** Mauricio Ceballos, Yaqueline Llano, Andrea Salazar-Ospina, Juliana Madrigal-Cadavid, Daniel Pino-Marín, Pedro Amariles

**Affiliations:** I Universidad de Antioquia Facultad de Ciencias Farmacéuticas y Alimentarias Departamento de Farmacia MedellínAntioquia Colombia Universidad de Antioquia. Facultad de Ciencias Farmacéuticas y Alimentarias. Departamento de Farmacia. Medellín, Antioquia, Colombia

**Keywords:** Tramadol, supply & distribution, Good Dispensing Practices, Professional-Patient Relations, Education, Pharmacy, Drugstores, Pharmacy staff, Fiscalized Substances

## Abstract

**OBJETIVE:**

To evaluate the skills and practices of pharmacy staff during the dispensing of tramadol (drug with fiscalized substance) in drugstores and pharmacies in Medellin, Colombia.

**METHODS:**

A cross-sectional study was performed. The simulated patient technique was used. The main outcomes included the information provided on the dispensed drug (tramadol), the use of tools to provide information, and the information provided on drug precautions and use recommendations.

**RESULTS:**

We visited 305 drugstores and pharmacies. The average dispensing time was 2.3 min (SD 1.1 min). In nine drugstores and pharmacies (3.0%), tramadol was not dispensed because it was not in stock. In 17 drugstores and pharmacies (5.7%), the simulated patients were actively informed by the dispensing pharmacy staff; of these, 16 provided oral information and one provided oral and written information. Eight patients (2.7%) received information regarding tramadol use. However, 99% of patients were not informed about tramadol side effects such as dependence, sedation, or hypnosis, and none of the simulated female patients were informed on the precautions related to tramadol use during pregnancy or lactation.

**CONCLUSIONS:**

Communication skills and appropriate practices of pharmacy staff are critical to patient self-care. However, this study shows their difficulty in counseling about precautions and use recommendations of drugs with fiscalized substances. These outcomes could inform future studies focusing on the rational use of these drugs in drugstores and pharmacies. It is necessary to improve the pharmacy staff competencies through continuing education programs, to facilitate access to information and training.

## INTRODUCTION

In the Colombia, staff members of drugstores and pharmacies (ambulatory retail establishments) are often the first healthcare providers with whom a patient comes into contact before using a medication, mainly because drugstores and pharmacies are accessible. It is common for patients not to visit their doctors until their health problems worsen. Both drugstores and pharmacies are pharmaceutical establishments dedicated to selling allopathic, herbal, homeopathic products, cosmetics, personal hygiene products, medical devices, and dietary supplements. According to data on the occupational characterization of the pharmaceutical sector in Colombia, in 2003, there were 15,208 drugstores and pharmacies; it is estimated that this number grew in 60% until 2016^1^ . Drugstores and pharmacies may play an important role in improving pharmacotherapy and patient outcomes, promoting rational use of drugs and reducing healthcare costs^2 , 3^ .

The dispensing process is the delivery of one or more medications to a patient, with information about their proper use. In Colombia, regulations have established that the minimal information that must be offered concerns storage conditions, drug reconstitution for oral administration, dose measurement, common side effects, precautions, recommendations for use, and the importance of treatment adherence^4^ . Although the dispensing process seems simple, it requires adequate time and can be affected by multiple factors such as training and knowledge of the pharmacist, professional compensation, competence, and communication skills and practices^5^ . In this sense, pharmacy staff should undergo continuing education related to pharmacotherapeutic follow-up, dispensing, health education, and patient counseling, within their regular activities, to help obtain the best health outcomes for patients^6^ .

Drugs are used to prevent, diagnose, and cure diseases. However, inadequate drug use may cause health problems due to adverse drug effects, ineffectiveness, and increased health care costs^7 , 8^ . Many drugs with fiscalized substances are indicated to treat pain, obstetric emergencies, and mental and neurological disorders^9^ . The dispensation of these drugs without a valid medical prescription can cause drug abuse, addiction, overdose, and even death^10^ . Some studies have shown that the most commonly dispensed fiscalized substances in drugstores and pharmacies are amitriptyline (91.4%), tramadol (90%), and trazodone (60%). In addition, around 60% of drugstores and pharmacies does not have qualified personal as part of their pharmacy staff. Their perception regarding deficiencies and needs in the correct dispensation of fiscalized substances may be inadequate; a high percentage of pharmacy staff displays educational flaws centered on the correct use of drugs^11^ .

The simulated patient technique employs a person trained to go to a pharmacy or drugstore, with the same characteristics of a real patient, to evaluate aspects of customer service provided by the pharmacy staff^12^ . Drugstores and pharmacies are ideal for this type of observation; moreover, research can be performed in real-time, since pharmacy staff can be accessed directly, without prior appointments, unlike other health professionals. In Colombia, no information is available related to skills and practices of pharmacy staff in dispensing drugs containing fiscalized substances in drugstores and pharmacies. Therefore, the objective of this study was to evaluate the skills and practices of pharmacy staff during the dispensing process of tramadol (drug with fiscalized substance) in drugstores and pharmacies in Medellin and Metropolitan Area of the Aburra Valley.

## METHODS

### Study Design

A cross-sectional study was performed between April and June 2018 to evaluate pharmacy staff skills and practices regarding the dispensation of fiscalized substances in drugstores and pharmacies.

### Selection of Drugstores and Pharmacies

The sample consisted of 305 drugstores and pharmacies that were randomly selected in the Medellin and Metropolitan Area of the Aburra Valley (Bello, Itagui, Envigado, Sabaneta, Caldas, Copacabana, Girardota, and La Estrella), in Antioquia, Colombia. Drugstores and pharmacies were invited to participate in the study in two commercial meetings, but the pharmacy staff did not know the research methodology for data collection. Drugstores and pharmacies were eligible for inclusion in the study if they dispensed drugs containing fiscalized substances. During these meetings, the consent of the pharmacy staff was obtained. Drugstores and pharmacies or pharmacy staff did not receive any compensation for participating in the study. The random selection was determined using a computer-generated table of random numbers and placed in a numbered form, designed by a person who did not participate in the study, using Microsoft Excel® (version 2016, Microsoft®).

### Simulated Patient Scenario

A simulated patient visited the drugstores and pharmacies with a medical prescription for tramadol (100 mg/mL), with a recommended dose of 10 mL drops (10 drops/8 hours), indicated for a week-long episode of back pain associated with physical exertion. Tramadol was selected for evaluation for the following reasons: a) it is an opioid drug (i.e., a drug with controlled substances); b) previous studies have shown that tramadol is the second most common drug dispensed to ambulatory patients with or without a medical prescription^11^ ; c) it has a high probability of being used incorrectly, with a high incidence of abuse and dependence^15 , 16^ ; and d) a large number of studies and academic reports have focused on tramadol. However, more efforts are needed to clarify the abuse potential and safety profile of tramadol^17^ .

### Simulated Patient Protocol and Training

Twenty students of the Technology in Pharmacy Regency at the University of Antioquia enrolled as simulated patients, with an average age of 20 years old. An eight-hour training course was conducted with the 20 students to instruct them on passive patients (no questions or comments were made during the process, except if the pharmacy staff asked questions or provided information). During the training, a dispensing process was simulated (the simulated patient was using tramadol by prescription for the first time). The training was carried out in two different sessions:

### Session 1

The purposes of this session were a) to explain the process; b) to discuss matters related to confidentiality agreements and informed consent. The researchers supplied a description of a case and provided training and questionnaire (checklist) manuals. The simulated patients had the task of reading and reviewing the manuals as well as the simulated case and they were in constant contact with the researchers.

### Session 2

The purposes of this session were a) to provide a general review of the process and to clear up doubts and comments; b) to review checklist items one by one to clear up doubts and clarify terms; c) individual training of each simulated patient in the performance of their role. Also, feedback on the most relevant aspects of the performance was received and discussed.

### Instrument and Techniques for Data Collection:

A questionnaire was constructed and applied by the simulated patients to evaluate 1) the dispensed drug; 2) the use of tools to provide information by the pharmacy staff; and 3) the information provided on drug precautions and recommendations. To guarantee data quality and validity, a researcher accompanied the simulated patients to the drugstores and pharmacies, but did not enter during the interaction, thus validating the process and data storage. The simulated patient completed the standardized questionnaire immediately after leaving the pharmacy to minimize recall bias risk.

### Data Analysis

All data were analyzed using the software Statistical Package for Social Sciences (SPSS) version 23 for Windows. The characteristics of the pharmacies, pharmacy staff, and tramadol dispensing are described as proportions and ranges.

### Ethics Approval

Ethics approval was obtained from the Ethical Committee of the Public Health National School of the University of Antioquia (Colombia). The data were kept anonymous.

## RESULTS

We visited 305 drugstores and pharmacies distributed as follows: 176 (57.7%) in Medellin and 129 (42.3%) in the surrounding metropolitan area. In 296 (97%) of the drugstores and pharmacies, tramadol was dispensed, while nine establishments did not have tramadol in stock. The average cost of tramadol was $4,900 COP (SD $1,070; minimum $1,800, maximum $10,000). The average dispensing duration was 2.3 min (SD 1.1 min; minimum 1 min, maximum 7 min).

Out of the 296 drugstores and pharmacies where tramadol was dispensed, the simulated patients were actively informed by the pharmacy staff in 17 drugstores and pharmacies (5.7%); only one of them recommended non-pharmacological measures and the use of over-the-counter drugs, like acetaminophen, for back pain management. The information that the pharmacy staff provided, orally and in writing, was not part of any physical leaflet or educational material related to tramadol ( Table 1 ).

**Table 1:** About the dispensed drug and tools used to provide information (n = 296).

		Yes n (%)	No n (%)
*About the dispensed drug*			
Tramadol was correctly dispensed according to the prescribed dosage and concentration		296 (100)	0
The pharmacy staff dispensed a different drug		0	296 (100)
The pharmacy staff actively provided information and then dispensed tramadol (the simulated patient did not have to ask any questions)		17 (5.7)	279 (94.3)
Tramadol was dispensed without informing the patient		279 (94.3)	17 (5.7)
The pharmacy staff recommended the use of a complementary over-the-counter drug (acetaminophen, naproxen, etc.), or non-pharmacological measures for back pain		1 (0.3)	295 (99.7)
*Use of tools to provide information*			
The information provided by the pharmacy staffwas:	Only oral	16 (5.4)	280 (94.6)
	Only written	0	296 (100.0)
	Oral and written (both)	1 (0.3)	295 (99.7)
The pharmacy staff delivered educational material such as flyers with information on the rational use of drugs and/or pain management		0	296 (100)
The pharmacy staff evaluated the satisfaction of the user/patient		0	296 (100)
The information provided by the pharmacy staff was clear and precise		8 (2.7)	288 (97.3)

In total, 80% (16) of the simulated patients were women; however, none were informed on the precautions related to tramadol use during pregnancy or lactation. Only 1.7% (5) of the simulated patients were correctly or moderately informed about precautions related to the possibility of dizziness and drowsiness. In 99% (293) of drugstores and pharmacies, the simulated patients did not receive information about the side effects of tramadol, such as dependence, sedation, or hypnosis, and none of the patients were informed on the precautions associated with alcohol consumption during treatment. Finally, 98.6% (292) of simulated patients were not informed on the importance of treatment adherence ( Table 2 ).

**Table 2:** About the information provided by the pharmacy staff (n = 296).

	Correctly informed n (%)	Moderately informed n (%)	Uninformed n (%)	Does not apply n (%)
*Information on the following precautions*				
Use of tramadol in women who are in the first trimester of pregnancy, who suspect they are pregnant or who are lactating	0	0	235 (79.4)	61 (20.6)^a^
Use in people who drive (car, motorcycle, bicycle) or who use heavy or high-risk equipment (construction, carpentry), due to the possibility of dizziness and drowsiness	1 (0.3)	4 (1.4)	291 (98.3)	0
Tramadol may cause dependence or addiction	2 (0.7)	1 (0.3)	293 (99.0)	0
Use of tramadol in people who suffer from seizures (epileptic seizures)	0	0	296 (100.0)	0
Use of tramadol with other drugs that generate sedation and hypnosis (drugs for depression, anxiety, sleep disorders)	2 (0.7)	1 (0.3)	293 (99.0)	0
Avoid alcohol consumption during tramadol treatment	0	0	296 (100.0)	0
*Information on the recommendations for use:*				
Dilute tramadol in a glass of water before swallowing	2 (0.7)	3 (1.0)	291 (98.3)	0
Administer tramadol independently of meals (before, during or after eating)	2 (0.7)	1 (0.3)	293 (99.0)	0
Do not use tramadol for a long period of time	2 (0.7)	1 (0.3)	293 (99.0)	0
Adequate storage of tramadol in the home, especially due to the risk for family members and others	0	0	296 (100.0)	0
The importance of treatment adherence	0	4 (1.4)	292 (98.6)	0

^a^ Male simulated patients.

According to Good Dispensing Practices^18^ , the simulated patients evaluated the dispensing process using an evaluation scale to rate the process from bad to excellent. Two (0.7%) simulated patients assessed it as very good, six (2.0%) as good, nine (3.0%) as regular, and 279 (94.3%) as bad. None of the simulated patients evaluated the dispensing process as excellent.

## DISCUSSION

The present study evaluated the dispensing skills and practices in real conditions of the pharmacy staff of drugstores and pharmacies in Colombia. The evaluated indicators were the average dispensing duration, tramadol stock availability, drug dispensing process, the use of tools to provide information, the discussion of precautions, recommendations on acceptable drug use, and the dispensing quality process, according to Good Dispensing Practices^18^ .

The effectiveness and profitability of drugstores and pharmacies are linked to opportunity, time efficiency, and the availability of drugs requested by patients. Therefore, an important indicator is the duration of care; in this study, the duration of the dispensing process was, on average, 2.3 min, which is somewhat higher than in other studies performed in Brazil, Sudan, Nigeria, and Bangladesh (< 1 min), Tanzania and India (1.3 min), and Nepal (1.4 min)^19 - 22^ , but similar compared with reports from Turkey, Vietnam, and Iran (2.4–2.8 min)^5 , 23 , 24^ . However, according to the World Health Organization (WHO) recommendations, this is not an adequate duration for drug dispensing and counseling the patient; they recommend that pharmacists spend at least 3 min talking to each patient^25^ . This result is not surprising due to the limited information and counseling supplied by pharmacy staff in real practice; that only around 35% of the pharmacy staff have a technological degree (Technology in Pharmacy Regency), and only 5% have a professional degree (Pharmaceutical Chemistry)^11^ . This demonstrates the need to develop continuing education programs focused on improving the competence of the pharmacy staff.

Another aspect important in the dispensing process is quality. This study demonstrated that the dispensing process in drugstores and pharmacies in Medellin and the surrounding metropolitan area is poor. Dispensing is a process that ends with a patient leaving a drugstores and pharmacies with a defined quantity of medication and instructions on how to use the drug. Therefore, during dispensation, the pharmacy staff should provide enough information to ensure that the patient will safely and appropriately use the medication^26^ . In this study, the information needed to use tramadol properly was not offered spontaneously, and requests that the patient provide additional information were minimal. It is possible that this was due to insufficient knowledge of therapeutic concepts, poor collection of patient information, and deficiencies in communication skills and practices of pharmacy staff.

Tramadol has been shown to have a risk of addiction with chronic use and it is commonly associated with side effects such as nausea, vomiting, sweating, fatigue, sedation, and dry mouth^27 , 28^ . The results obtained from the simulated patients indicate that the great majority of pharmacy staff did not consider the precautions associated with the use of tramadol. Therefore, they did not provide information that could help patients prevent or reduce safety problems while using tramadol. Reports show that most physicians assume that the pharmacist will inform patients during the dispensing process^29^ . Equally, the literature recommends providing information that can change patients’ habits and improve their health, such as recommending non-pharmacological treatments^29^ . In this study, none of the simulated patients received information on non-pharmacological therapeutic alternatives or recommendations to take tramadol diluted in water, to administer it independently of meals, not to use it for a long period, and how to store it at home.

The quality of the dispensing process is associated with the perception of the pharmacy staff regarding their responsibility toward the rational use of drugs and with their workload. Some studies suggest that imposing an excessive workload on pharmacy staff reduces the time spent with each patient and leads to less control over doctor prescriptions; this should be optimized^31^ . Additionally, a competent and capable workforce is essential for all health care professions. The capacity to improve therapeutic outcomes, patient quality of life, scientific advancement, and the enhancement of public health are dependent on a competent foundation^32^ . For the WHO and the International Pharmaceutical Federation (FIP), competencies are characteristics that people reveal when they perform a task or a job and they are related to a successful performance of an activity. These competencies are developed through education, training, and experience^2^ . Finally, in drugstores and pharmacies, many prescription medicines are sold without medical prescription—for example, 80% of all sold antibiotics were dispensed without a prescription^33^ , and 82% of drugs with fiscalized substances were sold without a prescription^11^ . Overall, these retail establishments do not meet the minimum international standard for ambulatory care pharmacy practices^34 , 35^ ; such unfavorable findings of poor dispensing practices occurred mainly in establishments whose pharmacy staff did not have pharmaceutical training—what usually happens is the simple delivery or sale of drugs and not actually dispensing. It is necessary to find solutions, among them educational programs to minimally contribute to rational use of drugs and patients’ health and outcomes.

In this context, pharmacy staff members of Colombian drugstores and pharmacies need to improve the required competencies as to contribute to the quality of medication use, minimize medication errors, and help outpatients to better manage their medicines. Continuing education programs, communication, and relationships with other health professionals are key-factors to improve these competencies and, consequently, to achieve optimal patient medication management. Clinical trials are now seeking to establish whether a continuing education program for correct use of drugs with fiscalized substances improves the skills of the pharmacy staff concerning these drugs^36^ .

### Limitations

This study used the simulated patient methodology to evaluate aspects of customer service provided by the pharmacy staff. This methodology has been assessed for validity and reliability in pharmacy practice research^14^ . The simulated patients received formal training and were evaluated before the visits to guarantee the quality of information. Furthermore, a researcher accompanied the simulated patients on all visits to the drugstores and pharmacies. The same researcher ensured that the simulated patients completed the questionnaire immediately after each visit, thus avoiding common biases in this type of study. It is important to note that none of the simulated patients were detected by the pharmacy staff, which suggests that they were well-trained and reliable. One possible limitation of our study is that the visits were not audio recorded to minimize the possible biases associated with the collected information, as recommended by some systematic reviews^14^ .

## CONCLUSIONS

With a simulated patient technique, it was possible to evaluate the skills and practices of pharmacy staff when dispensing drugs with fiscalized substances, which revealed major flaws in the provided information. Only a small part of the research subjects provided information or education regarding the correct use of tramadol. Important gaps were identified in the information provided by the pharmacy staff on the proper use of drugs with fiscalized substances, health promotion, and risk prevention. This should be an opportunity for improvement that must be renewed over time. Pharmacy staff must undergo continuous education so that dispensation is the most complete and accurate as possible. These outcomes can be used in future studies focusing on rational drug use, especially regarding fiscalized substances and competencies of pharmacy staff.
